# Endoscopic Raman Spectroscopy for Molecular Fingerprinting of Gastric Cancer: Principle to Implementation

**DOI:** 10.1155/2015/670121

**Published:** 2015-05-27

**Authors:** Hyung Hun Kim

**Affiliations:** Department of Internal Medicine, College of Medicine, The Catholic University of Korea, Seoul, Republic of Korea

## Abstract

Currently, positive endoscopic biopsy is the standard criterion for gastric cancer diagnosis but is invasive, often inconsistent, and delayed although early detection and early treatment is the most important policy. Raman spectroscopy is a spectroscopic technique based on inelastic scattering of monochromatic light. Raman spectrum represents molecular composition of the interrogated volume providing a direct molecular fingerprint. Several investigations revealed that Raman spectroscopy can differentiate normal, dysplastic, and adenocarcinoma gastric tissue with high sensitivity and specificity. Moreover, this technique can indentify malignant ulcer and showed the capability to analyze the carcinogenesis process. Automated on-line Raman spectral diagnostic system raised possibility to use Raman spectroscopy in clinical field. Raman spectroscopy can be applied in many fields such as guiding a target biopsy, optical biopsy in bleeding prone situation, and delineating the margin of the lesion. With wide field technology, Raman spectroscopy is expected to have specific role in our future clinical field.

## 1. Introduction

In the era of minimally destructive treatment, the early diagnosis is more important than anything. Diagnosis and treatment for gastric cancer is also following this innovation. Gastric cancer is one of the most common cancer in the world and second most common cause of cancer-related deaths [1–3]. When gastric cancer is detected in an advanced stage, the 5-year survival rate is low: 10%–20% [4]. However, the 5-year survival rates of patients with early gastric cancer, limited to the mucosa or the submucosa, were 99% and 96%, respectively [3, 5]. Introducing endoscopic submucosal dissection (ESD) changed the paradigm of gastric cancer treatment. ESD currently has settled as the standard treatment for patients with a lower mortality risk from metastasis as compared to that from surgery [6]. Thus, early diagnosis and early resection have enormous influence on the quality of life and saving stomach, as well as the survival of patients with gastric cancer. Considering recent advancement of therapeutic endoscopic practice, it cannot be more important to emphasize accurate diagnosis of small early lesions.

The advance of optic technology has led the remarkable evolutions in endoscopic images. Currently, the resolution power of white light endoscopy is as high as to detect objects 10–71 microns in diameter, compared with the naked eye which can discriminate objects 125–165 microns in diameter [7]. Adding to this, enhancement technologies including surface enhancement, color enhancement, and edge enhancement were efficiently used to help finding out suspicious mucosal changes more easily [8]. High pixel image and special enhancement technologies are the basis of current endoscopic image system. Magnification endoscopy usually involves the use of a movable lenses that allows the endoscopist to zoom in on a small area of mucosa and magnify it up to 150 times [9]. Narrow Band Image added significant power to magnifying endoscopy for investigating microanatomy of gastrointestinal mucosa. The combination of two technologies produced clinically applicable outcomes for identifying gastric cancer [10–12]. Confocal laser endomicroscopy enables the endoscopist to obtain real-time* in vivo* histologic images, which is called optical biopsies for gastric cancer [13–15]. However, these advances were only focused on investigating morphologic changes not on biochemical or molecular analysis of target tissues. Currently, positive endoscopic biopsy is the standard criterion for gastric cancer diagnosis, but is invasive and impractical for screening high-risk patients who may have multiple suspicious lesions [16]. In addition, endoscopic biopsies are often small (about 3 mm in diameter) and maybe inconclusive both in making the diagnosis and in determining the type of gastric adenocarcinoma [17]. Additionally, adenomas were frequently revealed to be carcinomas after endoscopic resection [18–20]. Furthermore, it is not uncommon that pathologic results are not concordant among pathologists [21]. Considering this circumstance, there is a clear need for an objective imaging diagnostic tool, which relies on biochemical or molecular analysis of target tissues rather than an individual's assessment of cellular appearance.

Raman spectroscopy represents a unique optical vibrational technique based on the fundamental premise of inelastic light scattering for tissue diagnosis and characterization [22–24]. Taking advantage of the Raman spectroscopic ability of harvesting a wealth of fingerprint information from inter- and/or intracellular components such as proteins, lipids, and DNA in cells and tissue, Raman spectroscopy has shown great promise for histopathologic assessments at the biomolecular level [23–26]. Many investigations have been conducted to reveal the biomolecular fingerprint of gastric cancer compared with normal tissue. In this review, basic principle, biomedical fingerprinting, instrumentation, and clinical implication for gastric cancer of Raman spectroscopy were covered with specific physical explanation.

## 2. Principle of Raman Spectroscopy

**Figure 1:**
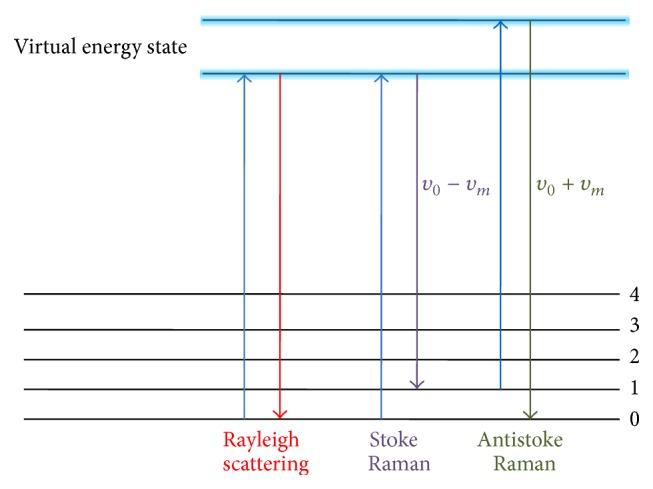
Three types of scattering: Rayleigh, Stoke, and Antistoke. Rayleigh scattering is a interaction that the excited molecule returns back to the same basic vibrational state and emits light with the same frequency *υ*_0_ as an excitation source. Raman scattering occurs when a photon with frequency *υ*_0_ is excited by Raman-active molecule. Stoke frequency is generated by the part of the photon energy is transferred with frequency *υ*_*m*_ and the resulting frequency of scattered light is diminished to *υ*_0_ − *υ*_*m*_. Antistoke frequency is released when a photon with frequency *υ*_0_ is excited by a Raman active molecule with already excited vibrational state. The energy level goes up to *υ*_0_ + *υ*_*m*_. Finally released frequency of scattered light from high virtual energy state to the ground state coincides with *υ*_0_ + *υ*_*m*_.

Raman spectroscopy is a spectroscopic technique based on inelastic scattering of monochromatic light, usually from a laser source [27]. Inelastic scattering is a phenomenon that the frequency of photons changes upon interaction with a sample. When a molecule is excited by the photons of the laser light and then reemitted photons, frequency of the reemitted photons is shifted up or down compared with original monochromatic frequency. This is called the Raman effect [28, 29]. This shift of frequency provides unique information of molecules. Monochromatic laser light with frequency *υ*_0_ excites molecules and transforms them into oscillating dipoles. Such oscillating dipoles emit light of three different frequencies when they return to lower energy level [28]. Raman scattering separated into two types: stoke and antistoke. Stoke is a phenomenon that emitted frequency is lower than exciting frequency. A photon with frequency *υ*_0_ excited Raman-active molecule which at the time of interaction is in the basic vibrational state. Part of the photon's energy is transferred to the Raman-active mode with frequency *υ*_*m*_ and the resulting frequency of scattered light is reduced to *υ*_0_ − *υ*_*m*_ (Figure 1). When an emitted frequency is higher than an excited frequency, this scattering is called as antistoke. A photon with frequency *υ*_0_ excited a Raman-active molecule, which, at the time of interaction, is already in the excited vibrational state. Excessive energy of excited Raman active mode is released, molecule returns to the basic vibrational state and the resulting frequency of scattered light goes up to *υ*_0_ + *υ*_*m*_ (Figure 1). Only about 0.001% of the incident photons generates Raman signal with frequencies *υ*_0_ ± *υ*_*m*_; approximately 99.999% of incident light undergo Rayleigh scattering, but this signal is not useful for molecular characterization; Rayleigh scattering is an elastic interaction between a photon and a molecule; a photon with the frequency *υ*_0_ is absorbed by a molecule with no Raman-active mode. After then, the excited molecule returns back to the same basic vibrational state and emits light with the same frequency *υ*_0_ as an excitation photon.

### 2.1. Biochemical Fingerprint by Raman Spectroscopy

**Figure 2:**
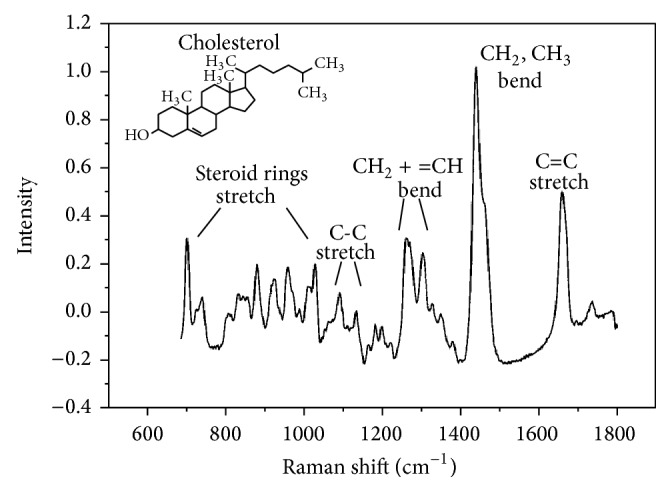
Near-infrared Raman spectrum. Near-infrared Raman spectrum of cholesterol indicating typical vibrational bands. The background has been removed by subtracting a fourth-order polynomial fit to the raw spectrum (adopted from Hanlon et al. Phys Med Biol 2000; 45: R1-59) [27].

**Figure 3:**
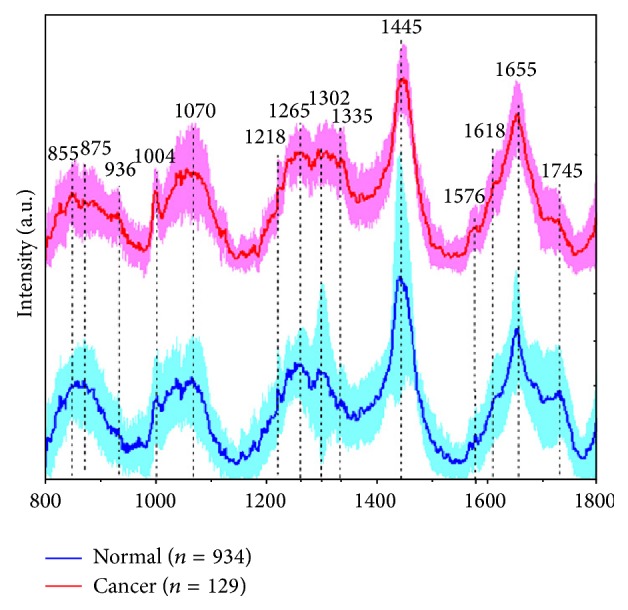
*In vivo* mean Raman spectra ±1 standard deviations of normal (*n* = 934), and cancer (*n* = 129) gastric tissue (adopted from Huang et al. Biosens Bioelectron 2010; 26: 383–389) [30].

Raman spectroscopy enables elucidating the biochemical characterization of a tissue through estimating the molecular specific inelastic scattering [27]. Raman spectroscopy needs a monochromatic laser illumination to sample tissue. Subsequently, collection and analysis of the scattered light is required. Photons in the incident laser light undergo inelastic collisions with molecules, causing an exchange of energy and therefore a change in frequency. The frequency change is dependent on the species of molecule. The shift is independent of the wavelength of excitation, which means that the energy shift is constant for each separate molecular species. The intensity of the signal is directly proportional to the concentration of the molecular constituents. Therefore, the Raman spectrum represents molecular composition of the interrogated volume providing a direct molecular fingerprint. Raman spectrum is the series of the scattered light intensity versus its change in frequency. Raman frequency shifts are conventionally measured in wave numbers (cm^−1^). The biological molecule of cholesterol represented by Raman spectroscopy is demonstrated in Figure 2 [27, 31]. Each band of scattered light of Raman spectrum is the characteristic of specific molecular vibrations, which, taken all together, are unique for cholesterol. For example, the characteristic Raman peak at 1440 cm^−1^ is caused by the CH_2_ and CH_3_ deformation vibrations, and the peak at 1670 cm^−1^ matches to C=C stretching vibrations. If a sample of biological tissue has cholesterol, these peaks will be observed in its Raman spectrum. Thus, the Raman spectrum can provide a “fingerprint” of a substance from which the molecular composition can be determined [27]. Fortunately, the majority of biological molecules are Raman active, each with their own fingerprint [32]. As a result, Raman spectroscopy is a very sensitive tool to subtle biochemical and molecular changes, which is crucial for differentiating tissues (Figure 3). Additionally, water, the predominant component of living tissue, gives a negligible Raman signal due to the limited change in polarisability of the –OH bond. This, thus, enables analysis of fresh, unprepared tissue, both* in vitro* and* in vivo* [33]. These properties make Raman spectroscopy potentially a very powerful diagnostic tool.

### 2.2. Basic Instrumentation

**Figure 4:**
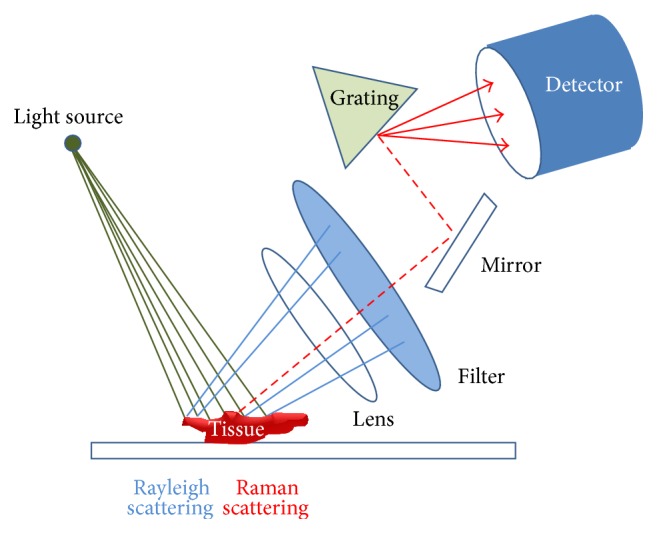
Schematic presentation of Raman spectroscopy instrument. When excitation light irradiates (green line), Rayleigh scattering (blue line) and Raman scattering (red dotted line) are released. Rayleigh scattering is filtered, and pure Raman scattering is detected.

**Figure 5:**
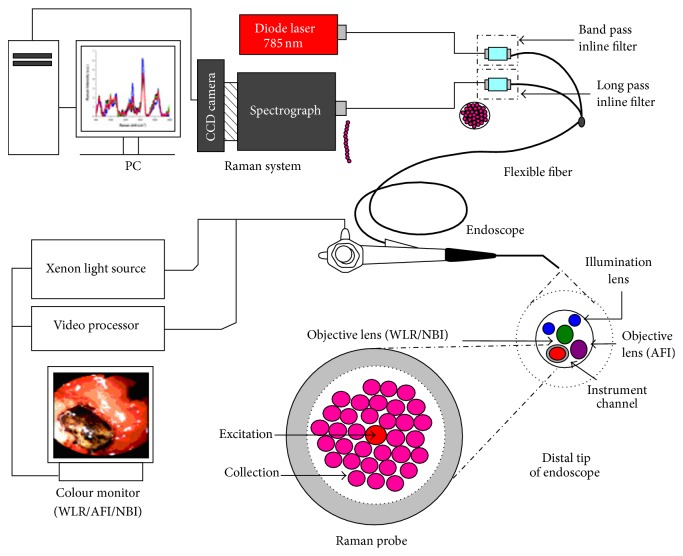
Schematic of the image-guided Raman endoscopy system for* in vivo* tissue Raman measurements during clinical gastroscopy. WLR, white light reflectance; AFI, autofluorescence imaging; NBI, narrow-band imaging (adopted from Huang et al. Biosens Bioelectron 2010; 26: 383–389) [30].

Spontaneous Raman scattering is very weak and special measures should be taken to distinguish it from the predominant Rayleigh scattering [27]. A Raman spectroscopy system typically consists of four major components: illumination source and system (laser), light collection optics, wavelength selector (filter or spectrophotometer), detector (photodiode array, charge coupled device or photomultiplier tube) (Figure 4). A lens collects the light, and a filter separates the Raman scatterings from the incident light. These shifted wavelengths then traverse the monochromator and detection system which measures their frequency. The specific frequencies of the shifted scattered light reveal the molecular structure of the sample material. Aside from Rayleigh scattering, fluorescence phenomenon is another hurdle to overcome for obtaining precise Raman scattering signal because the intensity of fluorescence is very strong and the signal spectrums overlap with Raman scattering. Using near infrared excitation is an excellent solution because it minimizes spectral disruption from tissue fluorescence [33]. Furthermore, near infrared light has less mutagenic effect and deeper penetration capability than other light sources. Ultraviolet-resonance Raman spectroscopy can be another option because fluorescence spectrum is separated from Raman spectrum when ultraviolet ray (wavelength < 270 nm) was illuminated [34, 35]. However, this method can cause mutagenesis and depth of tissue penetration is seriously limited. The combination of Raman spectroscopy and wide field endoscopic system used by Huang et al. was depicted in Figure 5.

## 3. Clinical Implementation

Huang et al. conducted many investigations to reveal the fingerprint of gastric cancer and dysplasia developing applicable algorithms [24, 30, 36–43]. This system consists of a 785-nm diode laser, a transmissive spectrograph with a Kaiser holographic grating, an near infrared-optimized back-illuminated, deep-depletion charge-coupled device detector (Princeton Instruments, Trenton, NJ, USA), and an in-house developed fiber-optic Raman probe that can effectively eliminate interference from fiber-optic fluorescence and silica Raman signals [44]. They fabricated a beveled fiber-optic confocal Raman probe coupled with a ball lens for enhancing* in vivo* epithelial tissue Raman measurements at endoscopy [25]. The confocal Raman probe design can be optimized for maximizing shallower tissue Raman measurements in epithelial tissue; in addition, the ratio of epithelium to stromal Raman photons collected using an optimized confocal Raman probe is approximately 19-fold higher than that using a volume-type Raman probe. The system acquired Raman spectra over the wave number range of 800 to 1800 cm^−1^, and each spectrum was acquired within 5s with light irradiance of 1.56 Wcm^−2^. The spectral resolution of the system is 4 cm^−1^. This group developed fully automated on-line Raman spectral diagnostics framework integrated with a multimodal image-guided Raman technique for real-time* in vivo* cancer detection at endoscopy [45]. Prior to developing on-line system, data-analysis has mostly been limited to post-processing and off-line algorithm development.

### 3.1. *Ex Vivo* Investigation

Teh et al. analyzed 76 gastric samples, 55 normal and 21 dysplasia, from 44 patients who underwent gastrectomy or endoscopic biopsies with clinically suspicious lesions. There are specific differences in Raman spectra between normal and dysplasia tissue, particularly in the spectral ranges of 1200–1500 cm^−1^ and 1600–1800 cm^−1^, which contained signals related to amide III and amide I of proteins, CH_3_CH_2_ twisting of proteins/nucleic acids, and the C=C stretching mode of phospholipids, respectively. The normal tissue showed Raman peak intensity at 875 cm^−1^ whereas the dysplasia demonstrated peak intensity at 1450 cm^−1^. Diagnostic algorithm based on principal components analysis (PCA) and linear discriminant analysis (LDA) yielded the diagnostic sensitivity of 95.2% and specificity 90.9% for separating dysplasia from normal gastric tissue [37]. Regarding differentiating gastric cancer from normal tissue, classification and regression tree, based on the recursive partitioning for classification of different subgroups in complex datasets, was introduced [36]. Raman peaks at 875 cm^−1^ and 1450 cm^−1^ were demonstrated in normal and dysplastic tissue, respectively, and the diagnostic sensitivity and specificity of learning dataset were 90.2% and 95.7%; the predictive sensitivity and specificity of the independent validation dataset were 88.9% and 92.9% (55 normal and 18 cancer samples) [36]. In 2013, Luo et al. demonstrated substantial difference among normal tissue, adenoma, and adenocarcinoma. They employed diverse statistical methods to develop effective diagnostic algorithms for classifying the Raman spectra of different types of* ex vivo* gastric tissues, including PCA, LDA, and naive Bayesian classifier (NBC) techniques. Compared with PCA-LDA algorithms, PCA and NBC techniques together with leave-one-out, cross-validation method provide better discriminative results of normal, adenoma, and adenocarcinoma gastric tissues, resulting in sensitivities of 96.3%, 96.9%, and 96.9%, and specificities of 93%, 100%, and 95.2%, respectively.

### 3.2. *In Vivo* Precancerous Lesion and Adenocarcinoma Study

The* in vivo* Raman spectroscopy study of gastric dysplasia and normal tissue was reported by Huang et al. combined with wide field endoscopy system, narrow band image, in 2010 [41]. There were significant differences between normal (*n* = 54) and dysplastic (*n* = 18) gastric tissue from 30 patients, particularly in the spectral ranges of 825 to 950, 1000 to 1100, 1250 to 1500, and 1600 to 1800 cm^−1^. Multivariate analysis analysis, based on PCA and LDA, together with the leave-one tissue site-out, cross validation on* in vivo* gastric Raman spectra yielded a sensitivity of 94.4% (17/18) and specificity of 96.3% (52/54) for distinction of gastric dysplastic tissue [41].

**Figure 6:**
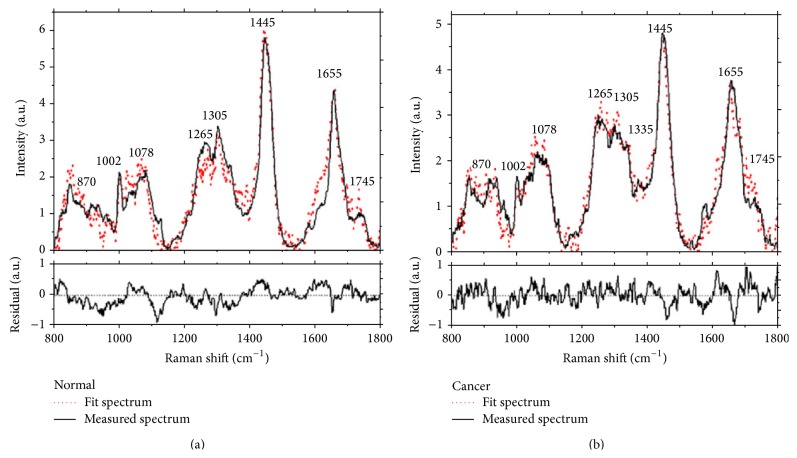
Comparison of* in vivo* gastric Raman spectra measured with the reconstructed tissue Raman spectra through the employment of the eight basis reference Raman spectra: (a) normal, and (b) cancerous gastric tissue. Residual of the fits are also shown on the same scale in each plot (adopted from Huang et al. Biosens Bioelectron 2010; 26: 383–389) [30].

The first* in vivo* study of gastric cancer was firstly presented in 2010 also. [30]. They revealed that the fit coefficients from albumin, nucleic acid, phospholipids and histones were found to be the most substantial features for construction of the diagnostic model, giving rise to an overall accuracy of 93.7%, sensitivity of 94.0% (110/117), and specificity of 93.4% (113/121) for* in vivo* discrimination of gastric cancer from normal gastric tissue: result from 1063* in vivo* Raman spectra from 238 tissue sites of 67 patients, 121 normal tissue and 117 gastric cancer tissue (Figure 6) [30]. Subsequent study, 238 gastric tissues from 67 patients, using ant colony optimization (ACO) integrated with LDA algorithm identified seven diagnostically important Raman bands in the regions of 850 to 875, 1090 to 1110, 1120 to 1130, 1170 to 1190, 1320 to 1340, 1655 to 1665 and 1730 to 1745 cm^−1^ provided a diagnostic sensitivity of 94.6% and specificity of 94.6% for distinction of gastric cancer [26].

The carcinogenesis of gastric cancer includes several premalignant cascade processes from chronic atrophic gastritis to intestinal metaplasia and dysplasia [46]. Bergholt et al. assessed the capability of Raman spectroscopy for multiclass elucidation of intestinal-type gastric carcinogenesis sequence* in vivo*. Raman spectroscopy integrated with semi-quantitative spectral modeling (e.g., DNA, lipids, glycoprotein, proteins and blood) reveals the progressive changes of biochemical constituents in gastric tissue associated with preneoplastic and neoplastic transformation [26]. Multiclass probabilistic partial least square-discriminant analysis (PLS-DA) diagnostic algorithms based on* in vivo* Raman spectra are able to identify normal mucosa with sensitivity of 75.88% and specificity of 87.21%; intestinal metaplasia with sensitivity of 46.67% and specificity of 87.55%; dysplasia with sensitivity of 83.33%; specificity of 96.80%, and adenocarcinoma with sensitivity of 84.91% and specificity of 95.57%. This study probed that Raman spectroscopy is a sensitive biomolecular probe for monitoring intestinal-type gastric carcinogenesis to realize early diagnosis and detection of precancerous lesions as well as gastric cancer.* In vivo* studies demonstrated that image-guided Raman endoscopy technique has promising potential for the noninvasive,* in vivo* diagnosis and detection of gastric cancer and dysplasia at the molecular level.

In clinical field, differentiating malignant ulcers from benign ulcers is quite important. However, this is not an easy task using conventional technique. Bergholt et al. demonstrated Raman spectroscopy can help clinicians in this point [39]. A total of 1102 Raman spectra were acquired from 71 patients with gastric ulcers (111 Raman spectra from benign ulcers, 67 Raman spectra from malignant ulcers, 924 Raman spectra from normal tissue). Distinctive spectral differences were observed in Raman spectra among three different tissues; particular spectral ranges were 800 to 900, 1000 to 1100, 1245 to 1335, 1440 to 1450 and 1500 to 1800 cm^−1^. Raman signal of a malignant ulcer is mainly associated with abnormal nuclear activity and decrease in lipids as compared to a benign ulcer. The partial least squares-discriminant algorithm together with leave on tissue site-out, cross validation technique yielded diagnostic sensitivities of 90.8%, 84.7%, and 82.1%, and specificities of 93.8%, 94.5%, and 95.3%, respectively, for classification of normal mucosa, benign ulcers, and malignant ulcerous lesions in the stomach [39].

**Figure 7:**
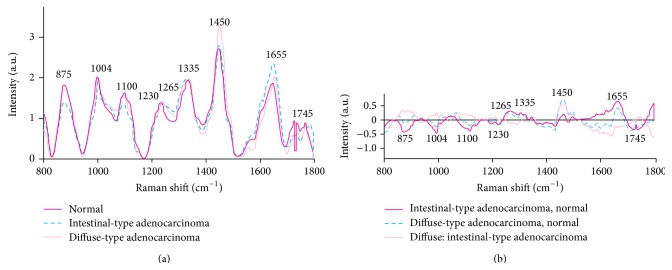
Comparison of mean near-infrared Raman spectra of 70 normal stomach samples, (a) 18 intestinal-type adenocarcinomas and 12 diffuse-type adenocarcinomas. (b) Difference spectra were calculated from the mean Raman spectra among the three gastric tissue types (adopted from Teh et al. Br J Surg 2010; 97: 550–557) [42].

The distinction between intestinal and diffuse types of gastric adenocarcinoma is clinically relevant and may influence treatment strategy [2]. The clinical potential of near infrared Raman spectroscopy for identifying different subtypes of gastric adenocarcinoma was reported by Teh et al. in 2010 [42]. There were significant differences in Raman spectra between normal stomach and the two gastric adenocarcinoma subtypes, particularly in the spectral ranges 850–1150, 1200–1500, and 1600–1750 cm^−1^, which contain signals related to proteins, nucleic acids and lipids (Figure 7). The predictive accuracies were of 88% with 92%. 94% for normal stomach, intestinal type adenocarcinoma, diffuse type adenocarcinoma, respectively. This result was reproduced by Kawabata et al.'s investigation [47].

### 3.3. Real Time on Line System

As above investigations presented, Raman spectroscopy is a notable method for understanding precancerous condition and adenocarcinoma in the stomach. However, data-analysis has been mostly performed after obtaining Raman spectrum in off line algorithm establishment before a fully automated on-line Raman spectral diagnostic system was developed for real-time* in vivo* cancer detection at endoscopy [45]. They tested this system with a total of 2748* in vivo* gastric tissue spectra (2465 normal and 283 cancer) from 305 patients [45]. Free-running optical diagnosis and processing time of no more than 0.5 s can be achieved, which is critical to realizing real-time* in vivo* tissue diagnostics during clinical endoscopic examination [45]. The optimized partial least squares-discriminant analysis (PLS-DA) models based on the randomly resampled training database provide the diagnostic accuracy of 85.6%, sensitivity of 80.5%, and specificity of 86.2% for the detection of gastric cancer. This on-line real time endoscopic Raman spectroscopy definitely opened a door for clinical application.

## 4. Conclusion

The goal of endoscopic Raman spectroscopy in the stomach is to accurate early diagnosis of gastric cancer which can be under or over diagnosed by conventional methods. Early accurate diagnosis provides a minimally invasive and minimally destructive treatment such as ESD, so the quality of patient is dramatically improved compared with surgical treatment. However, the accurate diagnosis of small obscure lesion is not easy in conventional method including forceps-biopsy. Several investigations demonstrated that adenoma was frequently revealed to be carcinoma after endoscopic resection within the range of 6% to 47% [18–20]. This discrepancy between forceps-biopsied specimen and post-resection specimen is inevitable limitation of forceps biopsy due to the lack of specimens [48–51]; especially, in the case of ESD, forceps-biopsy number is often significantly limited in order to prevent possible fibrosis that multiple forceps-biopsies can accompany. In this perspective, endoscopic Raman spectroscopy has a substantial potential in several points combined with wide filed technologies.

The most prominent feature of Raman spectroscopy is objective, reliable, and reproducible histologic estimation by molecular analysis making Raman spectroscopy different from other optical biopsy technologies such as confocal endomicroscopy. Confocal endomicroscopy is currently applied in usual clinical practice and produced several results regarding gastric cancer verification [52–54]. However, confocal endomicroscopy clearly needs long learning curve for maneuvering an endoscopy and probe to acquire high quality images although training for reading acquired picture may not be difficult [55, 56]. The presence of moving artifacts caused by breathing and heart beat disturb obtaining high quality images. Furthermore, there are similar problems that lurk in pathologic evaluation; interobserver discrepancy. Contrasting to other optical biopsy technologies, Raman spectroscopy automatically analyze the summation of specific molecular components in a target tissue and provide objective and reproducible diagnostic value through complicated algorithms [26, 30, 39, 41, 44, 45, 47]. This is a significant edge of Raman spectroscopy, which enables us to evade inter- and intra-observer discrepancies and to make consistent therapeutic plans for patients with same disease.

There are also limitations in Raman spectroscopy. First, Raman spectroscopy only identifies molecular features. Thus if two molecules have the same molecular features, they may be indistinguishable from each other, for instance, arachidonic acid and eicosapentaenoic acid. Second, fluorescence of impurities or of the sample itself can hide the Raman spectrum. Third, sample heating through the intense laser radiation can destroy the sample or cover the Raman spectrum.

Adding to diagnosing disease, Raman spectroscopy can be applied for understanding the margin of the cancer for obtaining the ideal resection area also. Following ESD, Raman spectroscopy can scan healed ulcer to confirm no cancer recurrence, especially when hypertrophic scar is noticed. This technology can guide accurate target biopsy instead of multiple forceps-biopsies. Furthermore, small erosions, frequently observed in elderly patients with anticoagulation therapy, might be easily elucidated with Raman spectroscopy without forceps-biopsy. For these applications, many prospective investigations would be necessary to verify its capability. Increased accuracy with developing updated algorithms and developing on-line real time automatic analysis system clearly opened a way to apply Raman spectroscopy for clinicians. Raman spectroscopy is expected to provide us with accurate, objective, and reliable diagnosis and may have potential to replace multiple forceps biopsies.
